# Rituximab in the Treatment of Interstitial Lung Diseases Related to Anti-Melanoma Differentiation-Associated Gene 5 Dermatomyositis: A Systematic Review

**DOI:** 10.3389/fimmu.2021.820163

**Published:** 2022-01-18

**Authors:** Chenjia He, Wenyu Li, Qibing Xie, Geng Yin

**Affiliations:** ^1^ Department of Rheumatology and Immunology, West China Hospital, Sichuan University, Chengdu, China; ^2^ Health Management Center, West China Hospital, Sichuan University, Chengdu, China

**Keywords:** dermatomyositis, melanoma differentiation-associated gene 5, interstitial lung disease, rituximab, targeting CD20

## Abstract

**Objective:**

The effectiveness of rituximab in anti-melanoma differentiation-associated gene 5 (MDA5) dermatomyositis (DM) with interstitial lung disease (ILD) has been explored only in isolated case reports and small series. This paper aims to review the current evidence regarding rituximab (RTX) use in the treatment of ILD related to anti-MDA5 DM (anti-MDA5 DM-ILD).

**Methods:**

We conducted a review by searching PubMed, Web of Science, Embase, and Cochrane for articles with information on patients with anti-MDA5 DM and RTX treatment, published until August 2021, in English language. The selected studies listed variation in chest high-resolution computed tomography (HRCT) and/or pulmonary function test (PFT) as a primary outcome, in patients with anti-MDA5 DM-related ILD after using RTX.

**Results:**

Of the 145 potentially eligible articles, 17 were selected. The information gathered from a total of 35 patients with anti-MDA5 DM-ILD was reviewed, including 13 men and 22 women. Patient age at onset was 47.60 ± 13.72 years old. A total of 11.43% (4/35) of the patients were found to have chronic ILD (C-ILD) and 88.57% (31/30) exhibited rapidly progressive ILD (RP-ILD). Most patients (29/30) had typical DM rashes. Prior to RTX administration, the majority of patients (27/35) were treated with medium- or high-dose glucocorticoids and at least one additional immunotherapeutic agent. With regard to RTX efficacy for ILD in anti-MDA5 DM, 71.43% (25/35) of the patients responded to treatment. Skin rash also improved in more than half of the patients after RTX treatment. The most common side effects were infections, reported by 37.14% (13/35) of the patients after using RTX.

**Conclusion:**

As a CD20 targeting drug, RTX is a promising therapeutic tool for anti-MDA5 DM-ILD, although the risk of infections should be considered before treatment. Further prospective controlled studies are required to evaluate the optimal RTX treatment regimen.

**Systematic Review Registration:**

https://www.crd.york.ac.uk/prospero/display_record.php?ID=CRD42021289714, identifier CRD42021289714.

## Introduction

Dermatomyositis (DM) is an autoimmune inflammatory disease that predominantly affects the muscles of the proximal extremities and the skin. DM is clinically heterogeneous both regarding patient symptoms and the severity of the disease. Myositis-specific autoantibodies (MSAs) are a class of recently discovered biomarkers that are associated to a unique clinical subset of myositis, and are therefore of* *great* *value in the classification of the disease, assessment of prognosis, and formulation of treatment plans (1). An important example of MSAs is the anti-melanoma differentiation-associated gene 5 autoantibody (anti-MDA5 autoAb). Patients positive for anti-MDA5 autoantibody (autoAb) typically exhibit cutaneous manifestations and mild or even no myopathy, but frequently are diagnosed with interstitial lung disease (ILD) (2). The prevalence of ILD in patients positive for anti-MDA5 autoAb has been estimated to be 50%–72.7% in reports from Europe and America (3–5) and 82%–100% in studies from Asia (6–10). In addition, the prevalence of patients that develop rapidly progressing ILD (RP-ILD) among the population of patients with anti-MDA5 DM-related ILD (anti-MDA5 DM-ILD) can be as high as 100% (11). More importantly, patients with anti-MDA5 DM usually have a high mortality rate due to relentless RP-ILD and lack of effective treatment. A recent study revealed that the 6-month survival rate of anti-MDA5 DM-ILD patients was only 33% even when treated with immunosuppressants (12).

Currently, there is no universal treatment for anti-MDA5 DM-ILD. Empiric treatment primarily focuses on glucocorticoid administration combined with the commonly used immunosuppressants - cyclophosphamide (CYC), calcineurin inhibitor (CNI) (12). However, there is still a large group of patients with anti-MDA5 DM-ILD who respond poorly to treatment with glucocorticoids and conventional immunosuppressants (13, 14). Hence, there is an urgent need to identify new treatment options for improving the therapeutic effect and prognosis.

CD20 is a transmembrane antigen selectively expressed on pre-B and mature B lymphocytes and is lost when B cells differentiate into plasma cells. In previous years, rituximab (RTX), a chimeric anti-CD20 monoclonal antibody, has been used in the management of B-cell malignancies (15), antineutrophil cytoplasmic antibody (ANCA)-associated renal vasculitis (16), rheumatoid arthritis (RA) (17), and systemic lupus erythematosus (SLE) (18). Although no guidelines on the treatment of myositis-related ILD have been published by the American College of Rheumatology (ACR) or by the European Alliance of Associations for Rheumatology (EULAR), RTX has also been used off-label in patients who did not respond to conventional therapy, based on a postulated pathogenetic role for B cells in anti-MDA5 DM-ILD. It is important to notice that the evaluation of therapeutic efficacy may be challenging in these conditions, since there are only a few case reports and small series in which RTX has been used in anti-MDA5 DM-ILD treatment.

The present paper aims to systematically review the currently available evidence regarding the use of RTX in anti-MDA5 DM-ILD.

## Methods

### Literature Search Strategy

**Figure 1:**
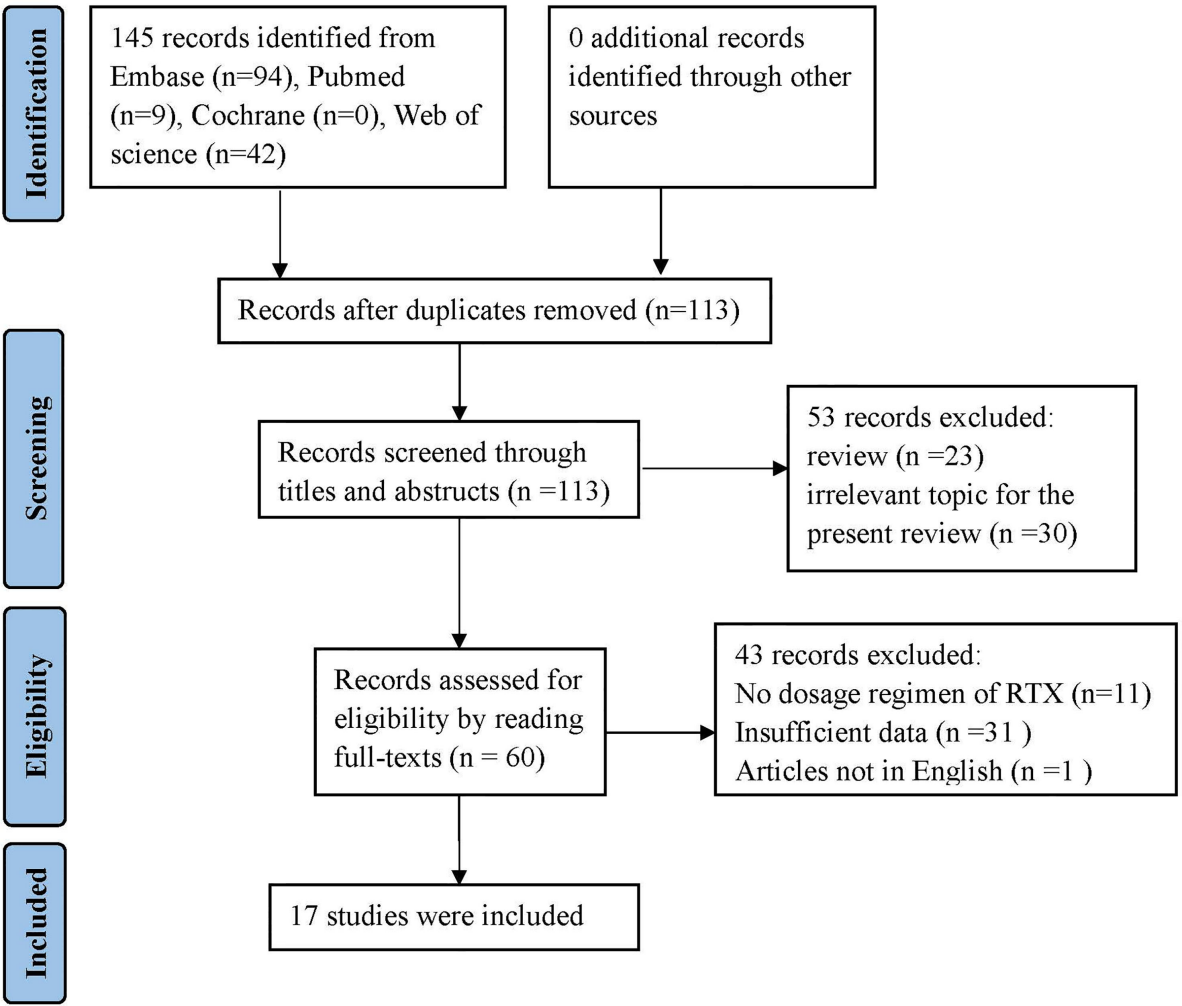
Flow diagram according to Prisma guidelines (20).

This systematic review was registered in PROSPERO (CRD42021289714) and performed according to the Preferred Reporting Items for Systematic Reviews and Meta-Analyses (PRISMA) recommendations (19). Two reviewers conducted a search on PubMed, Web of Science, Embase, and Cochrane databases, in an independent and simultaneous manner, for information on case reports, case series, and case-control studies of patients positive for anti-MDA5 DM and/or RTX treatment, reported until August 2021, in English language. The key retrieval terms included dermatomyositis, anti-MDA5, interstitial lung disease, Rituximab, and CD20 targeting. The detailed search strategy is documented in the supplementary file. The reference list of selected papers was also screened to identify additional studies to be included. **Figure 1** shows the flowchart of the paper selection process.

### Inclusion and Exclusion Criteria

Eligible patients in our review fulfilled the following criteria: i) met the Bohan and Peter criteria of DM (21) or Sontheimer’s criteria of CADM (22); ii) had positive anti-MDA5 antibody confirmed by immunoblotting assay or ELISA; iii) had confirmed interstitial lung diseases (ILD), defined as ground-glass changes and/or fibrosis noted on high-resolution computed tomography (HRCT) (23). iv) had receive RTX treatment. Subsequently, only cases with complete epidemiological data and therapeutic information were selected. Study designs were either randomized control trials (RCTs), cohort studies, case-control studies, case or case series. Exclusion criteria included suffering from malignancy or other overlapping rheumatic diseases, such as systemic lupus erythematosus (SLE) and systemic sclerosis (SSc).

### Data Extraction and Quality Assessment

Data recorded included age, sex, clinical manifestation (pulmonary involvement and/or extrapulmonary manifestations), laboratory data at disease presentation, and administered treatment (e.g., prednisolone, immunosuppressive agents, intravenous immunoglobulin, plasma exchange) previous to RTX administration were also recorded. Additional information collected included the RTX treatment schedule, RTX adverse effects, and clinical response to RTX. Two independent researchers assessed the methodological quality of all studies using the Newcastle-Ottawa Scale (NOS) criteria (24), classified according to the selection of the study groups, the comparability of the groups, and the ascertainment of the outcome. The classification scale ranges from 0 (poor methodological quality) to 9 (optimal methodological quality). Any discrepancies were resolved *via* discussion or consultation with a third researcher.

### Response Criteria

Patients were defined as responders if at least one of two criteria were met: i) ≥ 10% increase in forced vital capacity (FVC) and/or ≥ 15% increase in diffusing capacity of carbon monoxide (DLCO) (25); ii) improved outcome of lung imaging by either chest X-rays or HRCT (reviewed by a radiologist blinded to the design of the study) (23).

### Statistical Analysis

Data are expressed as the mean ± SD. Differences in quantitative parameters between two groups were assessed using the Mann-Whitney U-test. Fisher’s exact test was used to compare the trends between groups of qualitative variables. All statistical analyses were performed using Prism 8.3 software (GraphPad Software, La Jolla, CA, USA) and a two-sided p value of 0.05 or less was considered significant.

## Results

The bibliographic search conducted to identify cases of anti-MDA5 DM-ILD treated with RTX yielded 145 articles. After screening and eligibility check were conducted, 128 articles were excluded, as detailed in **Figure 1**. Of the 17 articles selected to be included in this review, 16 were case reports and case series, and one was a retrospective case-control study. Clinical data was extracted relating to 35 patients with anti-MDA5 DM-ILD meeting the inclusion criteria (23, 26–41). The demographic characteristics, clinical manifestations, laboratory data, treatment regimen, and outcomes are shown in **Tables 1**, **2**.

**Table 1:** Summary of the general characteristics of 35 patients with anti-MDA5 DM-ILD treated with RTX.

Characteristics	Patients with anti-MDA5 DM-ILD
Demographic	
Age at onset (years), mean ± SD	47.60 ± 13.72
Female, n (%)	22(62.86)
Asian, n (%)	33(94.29)
ILD, n (%)	35 (100)
C-ILD, n (%)	4(11.43)
RP-ILD, n (%)	31(88.57)
Cutaneous involvement	29/30
Heliotrope rash	20/30
Gottron’s papules or sign	24/30
Palmar papules	8/30
V-neck rash	8/30
Shawl sign	5/30
Mechanic hand	16/30
Ulceration	15/30
Myalgia	10/30
Muscle weakness	9/30
Fever	20/30
Arthralgia/arthritis	14/30
Raynaud’s phenomenon	8/30

RTX, rituximab; DM, dermatomyositis; MDA5, melanoma differentiation-associated gene 5; ILD, interstitial lung disease; RP-ILD, rapidly progressive interstitial lung disease; C-ILD, chronic interstitial lung disease.

**Table 2:** Clinical course of 35 patients with anti-MDA5 DM-ILD using RTX.

Patient no./Age at Entry, Years/Sex	Previous Therapy	RTX Treatment Schedule	Co-Intervention with RTX	Ferritin Levels at Admission/After RTX (ng/ml)	Outcome of Skin Rash	Outcome of Lung (Chest CT or PFT)	RTX Response/Outcome of Survival	Ref.
1/36/F	PSL, IVCY, CNI, MMF	375 mg/m^2^ i.v. at Day 0,Day 14	PSL 1mg/kg/d, MMF, CNI	77/10.8	Not improved	Chest CT improved, DLCO rose to 72.8% from 55% after RTX	Success/alive	1 (23)
2/59/M	PSL	375 mg/m^2^ i.v. at Day 0,Day 14	PSL 1mg/kg/d, IVIG, CNI	1119.3/110.7	improved	Chest CT improved, DLCO rose to 61.3% from 33.8% after RTX	Success/alive	
3/24/M	PSL, CNI, MTX, IVCY	375 mg/m^2^ i.v. at Day 0,Day 14	PSL 1mg/kg/d, CNI	749.8/366.5	improved	Chest CT improved after RTX	Success/alive	
4/41/F	PSL, IVCY, IVIG	375 mg/m^2^ i.v. at Day 0,Day 14	PSL 2mg/kg/d, IVCY	276.1/N.A.	Not improved	Chest CT deteriorated	Failure/died	
5/37/F	PSL, IVCY, MMF,IVIG	375 mg/m^2^ i.v. at Day 0,Day 14	PSL 2mg/kg/d, IVIG	1811/118	Not improved	Chest CT improved after RTX	Success/alive	
6/51/M	mPSL, AZA,IVCY	375 mg/m^2^ i.v. at Day 0,Day 14	PSL 2mg/kg/d, IVIG	1433.5/1169	improved	Chest CT deteriorated	Failure/died	
7/56/F	mPSL, IVCY, CNI	375 mg/m^2^ i.v. at Day 0, Day 14	PSL 1mg/kg/d, CNI	490.2/53.3	improved	Chest CT improved, DLCO rose to 71% from 36.6% after RTX	Success/alive	1 (23)
8/53/F	PSL, IVCY	100mg/w i.v. (for 4 ws)	PSL 1mg/kg/d, CNI	187.1/11.5	improved	Improvement in chest CT, PFT was initially not completed because of dyspnea. After RTX, DLCO rose to 70.1%	Success/alive	
9/28/M	mPSL, IVIG	100mg/w i.v. (for 4 ws)	PSL 1mg/kg/d, CNI	4676/24.4	improved	Improvement in chest CT, PFT was initially not completed because of dyspnea. After RTX, DLCO rose to 95.8%	Success/alive	
10/36/F	mPSL, CNI, IVIG	100mg/w i.v. (for 4 ws)	PSL 1mg/kg/d, CNI	1141.2/36.3	improved	Chest CT improved after RTX	Success/alive	
11/39/M	PSL, MMF, THD, IVCY	100mg/w i.v. (for 2 ws)	PSL 1mg/kg/d	1042.8/789	improved	Chest CT deteriorated	Failure/died	
12/49/F	PSL, MMF, CNI, IVCY, IVIG	In Ref.2, three in four patients use RTX 1g i.v. at Day 0, Day 14; one in four patients use RTX 500mg/w i.v. (for 4 ws)	PSL	N.A./23	improved	Chest CT improved, FVCrose to 67% from 39% after RTX	Success/alive	2 (26)
13/50/M	PSL, MMF, IVCY, CNI		PSL, CNI	N.A./260	improved	Chest CT improved, FVCrose to 105% from 76% after RTX	Success/alive	
14/38/M	PSL, MMF, CNI, IVIG		PSL	2844/902	improved	Chest CT unchanged, FVC rose to 121% from 94% after RTX	Success/alive	
15/48/M	PSL, CNI		PSL	N.A./170	improved	Chest CT improved, DLCO rose to 72% from 54% after RTX	Success/alive	
16/71/F	mPSL 1g×3d, followed by PSL 1mg/kg/d, CNI, IVCY, IVIg, PMX	350mg/m^2^/w i.v. (for 4 ws)	PSL, CNI	3149.8/253.1	improved	Chest CT improved after RTX	Success/alive	3 (27)
17/48/M	mPSL 1g×3d, followed by PSL 1mg/kg/d, CNI, IVCY	350mg/m^2^/w i.v. (for 4 ws)	PSL, CNI	781/186	improved	Chest CT improved after RTX	Success/alive	4 (28)
18/71/F	mPSL 1g×3d,followed by PSL 1mg/kg/d,CNI, IVCY	350mg/m^2^/w i.v. (for 2 ws)	PSL, CNI	507/1740	Not improved	Chest CT deteriorated	Failure/died	5 (29)
19/69/F	mPSL 1g×3d, followed by PSL 1mg/kg/d, CNI	350mg/m^2^/w i.v. (for 2 ws)	PSL, CNI, IVCY	219/1930	Not improved	Chest CT deteriorated	Failure/died	
20/57/M	PSL 1mg/kg/d, CNI, IVCY, PMX, MMF	550mg/w i.v. (for 4 ws)	PSL, CNI, MMF, IVIG	1178/continued to decrease	N.A.	Chest CT improved after RTX	Success/alive	6 (30)
21/49/F	PSL, HCQ	1g i.v. at Day0,Day 14	PSL, IVIG, CTX, CNI, EMCO	N.A./N.A.	N.A.	Chest CT deteriorated, Initial PFT results showed FVC and DLCO were 87.5% and 59% respectively	Failure/died	7 (31)
22/55/F	mPSL 500mg/week	375mg/m^2^/w (for 4 ws)	PSL 1mg/kg/d, CNI	1630/N.A.	improved	Chest CT improved, FVC rose to 88% from 60% after RTX	Success/alive	8 (32)
23/64/F	mPSL 500mg×3d, followed by mPSL 40-80mg/d, CNI, IVCY, IVIG	375mg/m^2^ (use twice)	mPSL 500mg×3d, followed by mPSL 40-80mg/d, CNI, IVIG	300/	N.A.	Chest CT deteriorated, Initial PFT results showed FVC and DLCO were 83.2% and 68.6% respectively	Failure/died	9 (33)
24/70/F	mPSL pulse therapy, followed by PSL, CNI, IVCY	375mg/m^2^/w (for 4 ws)	PSL, CNI	N.A./N.A.	improved	Chest CT improved after RTX	Success/alive	10 (34)
25/29/F	mPSL 1g×3d, followed by PSL 45mg/d, CNI, IVCY, PE	375mg/m^2^/w (for 4 ws)	PSL 40mg/d, CNI	77/decrease	improved	Chest CT unchanged	Failure/alive	11 (35)
26/50/F	PSL 25mg/d, MMF	1g i.v. at Day0,Day 14	PSL 12.5mg/d	N.A./N.A.	improved	Chest CT improved, DLCO rose to 56% from 42% after RTX	Success/alive	12 (36)
27/62/M	mPSL pulse therapy, followed by PSL 60mg/d, IVCY, CNI	500 mg i.v. at Day 3,Day 10, Day 17	mPSL pulse therapy, IVCY, CNI, TOF(5mg bid),PE(days 1,7, 8, 9)	6711.3/normal	N.A.	Chest CT improved after RTX	Success/alive	13 (37)
28/37/F	mPSL pulse therapy, followed by PSL 50mg/d	500 mg/w i.v.	mPSL pulse therapy, IVCY, CNI, TOF, PE	412.2/N.A.	N.A.	Chest CT improved after RTX	Success/alive	14 (38)
29/42/F	mPSL 1g×3d, mechanical ventilation	500 mg/w i.v. (only once)	None	169.4/N.A.	N.A.	Chest CT deteriorated	Failure/died	15 (39)
30/16/F	mPSL 1g×3d, followed by PSL1mg/kg/d, CNI	1g i.v. at Day 1, Day 15	IVCY, IVIG	2006/normal	improved	Chest CT improved, DLCO rose to 80% from 59% after RTX	Success/alive	16 (40)
31/44/M	GC	100 mg i.v. (only once)	GC, CNI, Other treatments are not mentioned	N.A./N.A.	N.A.	Chest CT improved after RTX	Success/alive	17 (41)
32/36/F	GC	100 mg i.v. (only once)	GC, CNI, Other treatments are not mentioned	N.A./N.A.	N.A.	Chest CT improved after RTX	Success/alive	17 (41)
33/55/F	GC	100 mg i.v. (only once)	GC, CNI, Other treatments are not mentioned	N.A./N.A.	N.A.	Chest CT improved after RTX	Success/died	
34/59/M	GC	100 mg i.v. (only once)	GC, CNI, Other treatments are not mentioned	N.A./N.A.	N.A.	Chest CT improved after RTX	Success/died	
35/37/F	GC	100 mg i.v. (only once)	GC, CNI, Other treatments are not mentioned	N.A./N.A.	N.A.	Chest CT deteriorated	Failure/died	

RTX, rituximab; PSL, prednisolone; mPSL, methylprednisolone; IVCY, intravenous cyclophosphamide; IVIG, intravenous immunoglobulin; CNI, calcineurin inhibitors; MMF, mycophenolate mofetil; MTX, methotrexate; AZA, azathioprine; TOF, tofacitinib; THD, thalidomide; PMX, polymyxin B hemoperfusion treatment; HCQ, hydroxychloroquine; PE, plasma exchange; w/ws, week/weeks; m, month; i.v., intravenous; N.A., not available; PFT, pulmonary function testing; FVC, forced vital capacity; DLCO, diffusing capacity of carbon monoxide; MAS, macrophage activation syndrome; HBV, Hepatitis B Virus; TB, tuberculosis; RFP, rifampin; success/failure, interstitial lung disease respond/did not respond to RTX treatment.

### Clinical Characteristics of Anti-MDA5 DM-ILD Patients Receiving RTX Treatment

The mean age at diagnosis was 47.6 ± 13.72 years. Approximately 62.86% (22/35) of the patients in our study were female and 94.29% (33/35) of patients were Asian. Patients with polymyositis (PM) and/or DM aggravated by ILD can be divided into two main clinical patterns: chronic (C-ILD) and rapidly progressive ILD (RP-ILD) (42), the latter being associated with poorer prognosis. In this study, all enrolled patients had ILD, from which 88.57% (31/35) had RP-ILD, and 11.43% (4/35) had C-ILD. Clinical manifestation data of 30 patients were obtained. Most patients (29/30) had typical DM rashes, such as heliotrope sign (20/30), Gottron’s sign (24/30), palmar papules (8/30), V-neck rash (8/30), shawl sign (5/30), mechanic hand (16/30), and ulceration (15/30). Myalgia and muscle weakness were observed in 10 and 9 patients, respectively. There were 20 patients with fever and 14 patients with arthralgia/arthritis. Raynaud’s phenomenon was observed in 8 patients (**Table 1**).

### Differential Treatment Regimen Across the Anti-MDA5 DM-ILD Patients

Prior to RTX administration, the majority of patients (27/35) in our study were treated with medium or high doses of glucocorticoids and at least one additional immunotherapy treatments, including intravenous cyclophosphamide (18/35, 51.43%), calcineurin inhibitor (18/35, 51.43%), intravenous immunoglobulin (8/35, 22.86%), mycophenolate mofetil (8/35, 22.86%), polymyxin B hemoperfusion treatment (2/35, 5.71%), plasmapheresis (1/35, 2.86%), methotrexate (1/35, 2.86%), azathioprine (1/35, 2.86%), hydroxychloroquine (1/35, 2.86%), and thalidomide (1/35, 2.86%). In this study, 19 patients were treated with two or more immunotherapy treatments. Remarkably, three patients received five treatment modalities. Approximately 42.86% (15/35) of patients were treated with the lymphoma schedule or lymphoma-like schedule (350–375 mg/m^2^ every 1 or 2 weeks) and 17.14% (6/35) of patients received the rheumatology schedule (500–1000 mg every 2 weeks). Other schedules included 500–550 mg RTX every week in 5 (14.29%) patients. In addition to the above conventional regimens, 9 anti-MDA5 DM-ILD patients were treated with a novel low-dose RTX regimen (100 mg per week)(**Table 2**).

### Efficacy of RTX for Anti-MDA5 DM-ILD Patients

Considering the efficiency of RTX treatment for ILD in anti-MDA5 DM patients, 71.43% (25/35) of patients presented a response to treatment (assessed by chest HRCT and/or PFT). In the C-ILD subgroup, 75% (3/4) of the patients presented a response to RTX, while 70.97% (22/34) of patients in the RP-ILD subgroup presented a response to RTX. Among the low-dose and conventional-dose RTX subgroups, the response rate to treatment was 77.78% (7/9) and 69.23% (18/31), respectively (**Table 3**). Ferritin data were collected from 24 patients in this study. The levels of ferritin were significantly higher in the RTX treatment responsive subgroup than in the non-responsive subgroup (P=0.0196)(supplementary Fig 1). Seventeen patients had decreased ferritin levels after RTX treatment. A decrease of anti-MDA5 autoAb titers observed in patients 17, 25 and 27 after RTX treatment, while data from other patients were not available. Considering the outcome of cutaneous involvement, 19 patients showed improvement, including reduced degree and size of skin rash or healing of skin ulcers.

**Table 3:** Effiacy and safety data of the 35 patients with anti-MDA5 DM-ILD treated with RTX.

	Pattern of ILD		RTX Treatment Schedule	
	C-ILD	RP-ILD	Low-Dose RTX	Conventional-Dose RTX
Total	4	31	9	26
Mean age, years (mean ± SD)	34.75 ± 11.30	49.26 ± 13.25	43 ± 10.46	49.19 ± 14.51
Female, n (%)	3 (75)	19 (61.29)	5 (55.56)	17 (65.39)
Response to RTX Treatment, n (%)	3 (75)	22 (70.97)	7 (77.78)	18 (69.23)
Infection, n(%)	N.A.	N.A.	5 (55.56)	8 (30.77)
Survival, n (%)	4 (100)	20 (64.52)	6 (66.67)	18 (69.23)

RTX, rituximab; DM, dermatomyositis; MDA5, melanoma differentiation-associated gene 5; ILD, interstitial lung disease; RP-ILD, rapidly progressive interstitial lung disease; C-ILD, chronic interstitial lung disease; N.A., not available.

### Safety of RTX in Treatment of Anti-MDA5 DM-ILD Patients

With regard to safety of RTX in anti-MDA5 DM-ILD patients, infusion reactions were not reported in any of the cases under study, and the most common side effects were infections. A total of 16 infection were reported by 13 patients, after RTX. Noteworthy, 56.25% (9/16) of the infection events were pulmonary infections, and more than half of them were caused by cytomegalovirus (CMV) (**Table 4**). The infection rate of patients in the low-dose and conventional-dose RTX subgroups was 55.56% (5/9) and 30.77% (8/26), respectively. The survival rates among patients with C-ILD and RP-ILD were 100% (4/4) and 64.52% (20/31), respectively. The survival rates of patients in the low-dose and conventional-dose RTX subgroups were 66.67% (6/9) and 69.23% (18/26), respectively (**Table 3**). The survival rates of patients in the RTX treatment responsive and non-responsive subgroups were 92% (23/25) and 10% (1/10), respectively (**Table 5**).

**Table 4:** Summary of infections in the patients comprised in the present study.

Infection Type	Number of Iinfection Events	Remarks
Upper respiratory infection	2	Occurred in patients 31, 32.
Pulmonary infection	9	Occurred in patients 4, 6, 11, 13, 14, 27, 32, 33, 34; Five of them were CMV infection.
Urinary tract infection	2	Occurred in patient 27, the other one is unknown.
Appendicitis	1	N.A.
Skin infection	1	Occurred in patient 12.
Herpes infection	1	N.A.

CMV, cytomegalovirus; N.A., not available.

**Table 5:** Clinical data between RTX treatment responsive and non-responsive subgroups in 35 patients with anti-MDA5 DM-ILD.

	RTX Treatment Responsive	RTX Treatment Non-Responsive
Total	25	10
Mean age, years (mean ± SD)	46.96 ± 13.68	49.20 ± 14.41
Female, n (%)	14 (56%)	8 (80%)
C-ILD, n (%)	3 (12%)	1 (10%)
RP-ILD, n (%)	22 (88%)	9 (90%)
Survival, n (%)	23 (92%)	1 (10%)

RTX, rituximab; DM, dermatomyositis; MDA5, melanoma differentiation-associated gene 5; ILD, interstitial lung disease; RP-ILD, rapidly progressive interstitial lung disease; C-ILD, chronic interstitial lung disease.

## Discussion

**Figure 2:**
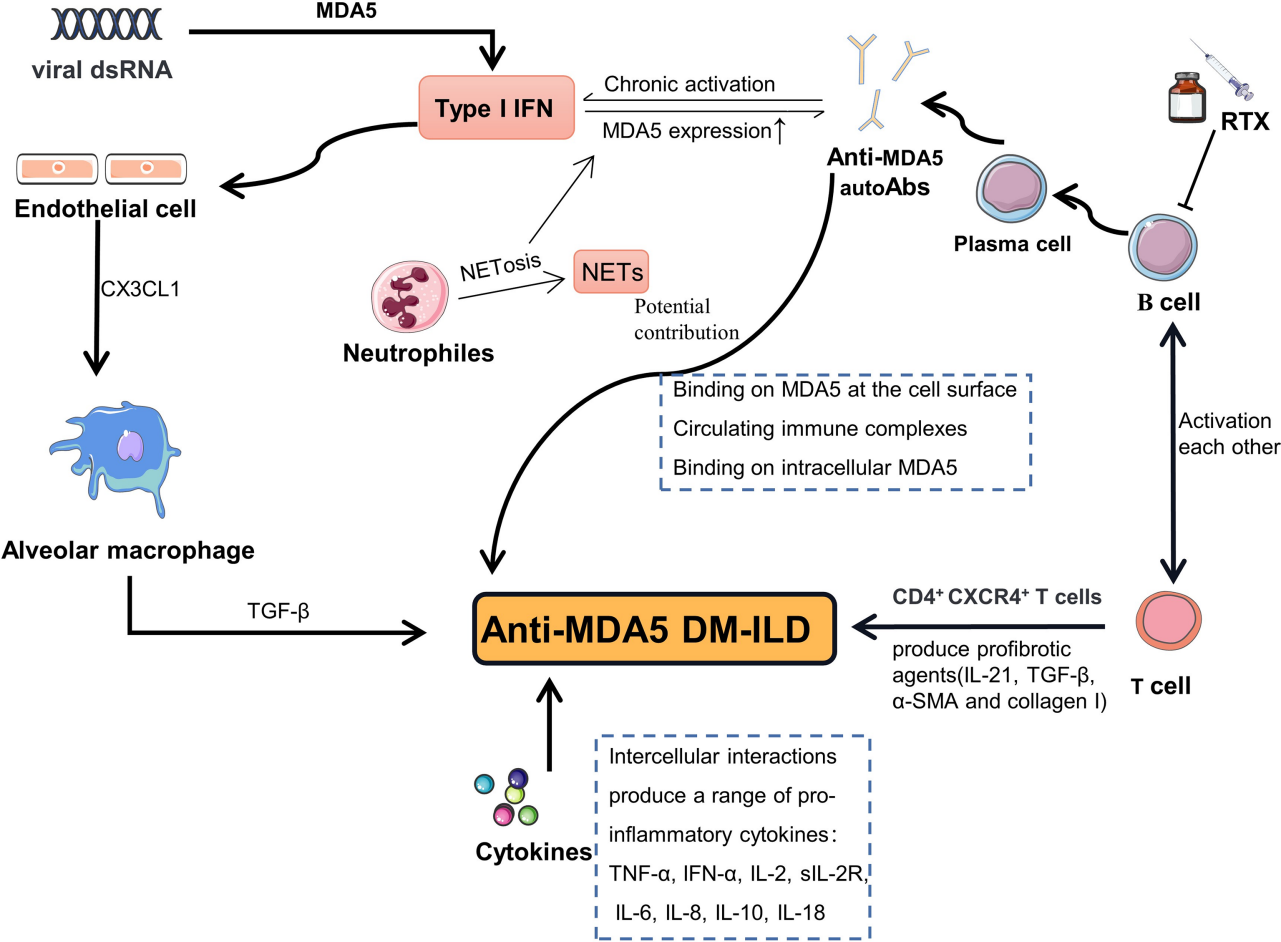
Schematic representation of the hypothesized pathogenesis in anti-MDA5 DM-ILD. MDA5 protein can recognizes viral dsRNA then activates IFN-I pathway. Activation of the IFN-I system promote the production of MDA5 and cause MDA5 overexpression. Abnormal accumulation of MDA5 protein may lead to a loss of immune tolerance, resulting in the production of anti-MDA5 autoAb. Anti-MDA5 autoAbs may potential contribute to the pathogenesis through binding to MDA5 on cell surface, forming immune complexes, and interacting with cytoplasmic MDA5. In the lungs, CX3CL1 can be produced by endothelial cells when exposed to IFN-I and induce recruitment of alveolar M2 macrophages. Local production of TGF-β by M2 macrophages directly promotes pulmonary fibrosis. CD4^+^CXCR4^+^ T cell subset in anti-MDA5 DM-ILD can produce profibrotic agents (TGF-β, α-SMA, collagen I, and IL-21). The process of activated neutrophils releasing NETs could expose MDA5 autoantigens. MDA5, melanoma differentiation-associated gene 5; DM, dermatomyositis; ILD, interstitial lung disease; autoAb, autoantibody; RTX, rituximab; dsRNA, double-stranded ribose nucleic acid; NETs, neutrophil extracellular traps; NETosis, neutrophil extracellular traps externalization process; IFN-I, type I interferon; TGF-β, transformation growth factor-β; α-SMA, α-smooth muscle actin.

The hallmarks of anti-MDA5 DM are the presence of autoantibodies targeting MDA5 and unique cutaneous features, as well as an elevated risk of ILD. The underlying pathogenesis is not yet fully understood. T and B lymphocytes (23, 43–47), neutrophils (48, 49), macrophages (50, 51), type I interferon (IFN-I) (52, 53) and “Cytokines storm” [which is similarity with SARS-CoV-2 infection (54)] were thought to be involved in the development of the disease (**Figure 2**). MDA5 protein, as a pattern recognition receptor (PRR), can recognizes viral double-stranded RNA (dsRNA) then activates IFN-I pathway (55) and induces the production of proinflammatory cytokines by the cell. Indeed, the aberrant activation of the type I interferon system has been demonstrated in anti-MDA5 DM in previous studies (52, 53). While MDA5 protein is also an IFN-inducible protein (56), so activation of the IFN-I system can further promote the production of MDA5 protein. Abnormal accumulation of MDA5 protein may lead to a loss of tolerance to MDA5, resulting in the production of anti-MDA5 autoAb. As for how anti-MDA5 autoAbs play their potentially pathogenic role is less well understood and is only briefly described here in light of a recent review on the subject (47). In pathological contexts, MDA5 is strong expressed in skin tissue of DM patients (57) and detectable in cytoplasmic, cell surface, and secretory vesicles in neutrophils (58). Hence, some scholars have speculated that anti-MDA5 Abs can bind to MDA5 on cell surface and induce an inappropriate activation of MDA5, resulting in chronic activation of the IFN-I pathway. Apart from binding to MDA5 expressed on cell surface, they suggested anti-MDA5 Abs could also form immune complexes with the MDA5 proteins released from apoptotic cell and interact with cytoplasmic MDA5, which may similar to other Abs described previously (59, 60). In the lungs, the chemokine CX3CL1 can be produced by vascular endothelial cells when exposed to IFN-I and induce recruitment of CX3CR1^+^ M2 macrophages (61, 62). Local production of transformation growth factor-β (TGF-β) by M2 macrophages directly promotes pulmonary fibrosis. Moreover, there is a CD4^+^CXCR4^+^ T cell subset in anti-MDA5 DM-ILD, which can produce profibrotic agents (TGF-β, α-smooth muscle actin (α-SMA), collagen I, and IL-21) (46). In addition, the process of activated neutrophils releasing neutrophil extracellular traps (NETs) could expose autoantigens that have the potential to break immune tolerance and lead to production of autoantibodies (63). In recent years, accumulating evidence suggested that B lymphocytes may play a critical role in the pathogenesis of anti-MDA5 DM-ILD. First, plasma cells that secrete antibodies derive from B lymphocytes, while multiple studies have already demonstrated that anti-MDA5 autoantibodies are closely related to disease activity and ILD in patients with anti-MDA5 DM (64, 65); Secondly, B cell activating factor (BAFF) is a member of the tumor necrosis factor (TNF) superfamily, playing a key role in the survival and balance of peripheral B cells and plasma cells. It not only promotes the maturation of B cells, but also regulates immunoglobulin class-switching (66, 67). Data from Kobayashi et al. showed that juvenile dermatomyositis (JDM) patients with a high titer of anti-MDA5 autoantibodies had higher levels of BAFF than those with low titers (68). Another study noted that higher levels of BAFF were detected in patients positive for anti-MDA5 autoantibodies, when compared to negative patients, and that the levels of BAFF correlated positively with the titers of anti-MDA5 autoantibodies. The same study also indicates that the BAFF level of anti-MDA5 DM patients aggravated by ILD was significantly higher than anti-MDA5 DM patients not suffering from ILD, and the level of BAFF was parallel with KL-6, an indicator of the severity of ILD, suggesting that patients with anti-MDA5 DM complicated with severe ILD tend to have higher levels of serum BAFF (52); Thirdly, a recent study by Shuang Ye et al. revealed that the peripheral percentage of CD4^+^CXCR4^+^T cells is relevant to the severity of ILD in idiopathic inflammatory myopathy (IIM), especially to anti-MDA5 DM-ILD. Furthermore, they confirmed that the circulating CD4^+^CXCR4^+^T cell subset expresses high levels of IL-21 (46), which can induce the differentiation of B cells into plasmablasts by binding to the IL-21 receptor on the surface of B cells (69).

Based on the possible causative role of B cells, targeting CD20 in the treatment of anti-MDA5 DM-ILD seems to be of great importance. Hence, RTX is empirically used as a therapeutic agent for patients with anti-MDA5 DM-ILD. All currently published studies on the efficacy of RTX in the treatment of anti-MDA5 DM-ILD are case reports, case series, or case-control studies. The present work offers a systematic review of the effects of RTX in 35 anti-MDA5 DM-ILD patients. The clinical response after using RTX for ILD could be defined by the improvement of chest HRCT and/or PFT. After RTX treatment, 71.43% (25/35) of patients responded positively, according to chest HRCT and/or PFT. According to the data here analyzed, anti-MDA5 DM patients with RP-ILD (22/31, 70.97%) had a lower rate of response to RTX than C-ILD (3/4, 75.0%) patients. We also observed that patients in the low-dose RTX subgroup (7/9, 77.78%) had a higher response rate than patients in the conventional-dose RTX subgroup (18/26, 69.23%). In addition, patients with anti-MDA5 DM usually exhibit typical cutaneous manifestations. Previous studies that assessed the efficacy of RTX for cutaneous lesions in anti-MDA5 DM patients have demonstrated different therapeutic effects (70, 71). In our study, skin rash improved in more than half of the patients that used RTX, whereas the rate of response to RTX treatment on cutaneous lesions in anti-MDA5 DM patients cannot be accurately calculated due to the unavailability of information on some cases. Further large-sample studies are required to evaluate the efficacy of RTX on the cutaneous lesions of patients with anti-MDA5 DM. Similarly, we observed that ferritin levels decreased in more than half of the patients, after RTX treatment. A study by Gono et al. showed that ferritin level is a poor prognostic factor in RP-ILD patients with anti-MDA5 DM, and also indicated that ferritin concentrations are useful for the evaluation of the response to treatment in patients with anti-MDA5 DM-ILD (72). This indicates that the condition of most patients in our study improved after RTX. Considering the results described above and the fact that all patients in our study received glucocorticoids and/or immunosuppressants prior to RTX treatment and had poor response to these drugs, RTX may be an effective treatment for anti-MDA5 DM-ILD resistant to glucocorticoids and multiple immunotherapies.

From a safety perspective, 37.14% (13/35) of patients developed infection after RTX therapy, and four patients died of pulmonary infection in our study. Although there seems to be an increased risk of clinical infections after RTX treatment, the rate of infection is poorly correlated with the types of immune diseases and is closely correlated with low hypogammaglobulinemia, neutropenia, CD4^+^ T cell dysfunction, etc. (73). Recently, a registry-based study estimated at 30% the efficacy and safety for RTX treatment in anti-synthetase syndrome, which was similar to the rate of infections after RTX administration in anti-MDA5 DM-ILD in the present review. In addition, 55.56% (5/9) of the patients in the low-dose subgroup were infected, which proved to be higher than the percentage of infections in the conventional-dose subgroup (30.77%) (8/26). This is most likely due to the fact that 6 patients who received low-dose RTX in Reference 17 were not included (due to lack of chest HRCT and/or PFT data), which led to biases in the results. Given that the response rate to RTX treatment of patients to in the low-dose subgroup (77.78%) was also higher than that of patients from the conventional-dose subgroup, and that this low-dose RTX regimen has been successfully used in several other immune diseases (74–76), we speculate that low-dose RTX (100 mg per week) may lead to a better therapeutic response than the conventional dose regimen. However, due to the limited number of patients in our study, whether low-dose RTX is recommended for anti-MDA5 DM-ILD remains to be verified in larger sample trials.

To our knowledge, the data on anti-MDA5 DM-ILD patients treated with RTX reviewed in this study were larger than those in previous studies, and we describe the detailed regimen used for RTX administration. In addition, we evaluated the changes in ILD through HRCT and/or PFT, providing more evidence for the efficacy and safety of RTX in the treatment of anti-MDA5 DM-related ILD. However, there are some limitations to this study. First, the statistical analysis of the results was limited by the small sample size, which is not easy to circumvent due to the rarity of the disease. Second, the appropriate timing of RTX administration was not demonstrated in the present study. Third, the information of anti-MDA5 autoAb titers before and after RTX treatment were not available in most cases. Finally, multiple immunosuppressants were administered prior to RTX therapy in most patients, which may prevent the attribution of improvement to RTX treatment alone.

In summary, this systematic review allows us to conclude that as a CD20 targeting drug, RTX has a good response in the treatment of ILD related to anti-MDA5 DM. Combined with the current evidence that B cells may be involved in the pathogenesis of this disease, we suggest that RTX could be a promising treatment for anti-MDA5 DM-ILD. Also, patients with anti-MDA5 DM-ILD often have a condition with worse prognosis and were treated with multiple immunosuppressants previously or simultaneously to RTX administration, the risk of infection, especially opportunistic infections, should be considered during the use of RTX, and a low dose of RTX (100 mg every week) may also be applied; In addition, belimumab, a human monoclonal antibody targeting BAFF, may also be considered as a candidate therapy for anti-MDA5 DM-ILD, since some cases in our study showed poor response to RTX treatment. Finally, further prospective controlled clinical studies are required to evaluate the status and optimal regimen of RTX in anti-MDA5 DM-ILD.

## Data Availability Statement

The original contributions presented in the study are included in the article/**Supplementary Material**. Further inquiries can be directed to the corresponding authors.

## Author Contributions

QX, GY, and CH conceived the study. CH designed the study forms. GY and QX guided this study. CH and WL searched the literature and screened the studies for inclusion, extracted data, and assessed methodological quality. CH, WL, GY, and QX organized and analyzed data. All authors drafted and revised the manuscript. All authors contributed to the article and approved the submitted version.

## Funding

This study is supported by Sichuan Science and Technology Program (Grant number 2021JDRC0045, 2021JDRC0169, 2021YJ0472 and 2021YFS0164), and the Clinical Research Incubation Project of West China Hospital, Sichuan University (Grant number 2019HXFH038 and 2021HXFH018).

## Conflict of Interest

The authors declare that the research was conducted in the absence of any commercial or financial relationships that could be construed as a potential conflict of interest.

## Publisher’s Note

All claims expressed in this article are solely those of the authors and do not necessarily represent those of their affiliated organizations, or those of the publisher, the editors and the reviewers. Any product that may be evaluated in this article, or claim that may be made by its manufacturer, is not guaranteed or endorsed by the publisher.
