# Supplementary material for: Rituximab in the Treatment of Interstitial Lung Diseases Related to Anti-Melanoma Differentiation-Associated Gene 5 Dermatomyositis: A Systematic Review
Source: Front Immunol. 2022 Jan 18;12:820163. doi: 10.3389/fimmu.2021.820163 (PMC8803653; doi:10.3389/fimmu.2021.820163)
Supplement: Supplementary file 1 [file DataSheet_1.zip › Supplementary Table 1.DOC]

**Web of science search strategy**

**Search Query  Results**

5

**(((#1) AND #2) AND #3) AND #4**

**42**

4

**(((((((((((TS=(Rituximab)) OR TS=(CD20 Antibody, Rituximab)) OR TS=(Rituximab CD20 Antibody)) OR TS=(Mabthera)) OR TS=(IDEC-C2B8 Antibody)) OR TS=(IDEC C2B8 Antibody)) OR TS=(IDEC-C2B8)) OR TS=(IDEC C2B8)) OR TS=(GP2013)) OR TS=(Rituxan)) OR TS=(CD20 targeting)) OR TS=(Targeting CD20)**

**59,760**

3

**(((((((((((((TS=(Lung Diseases, Interstitial)) OR TS=(Diffuse Parenchymal Lung Disease)) OR TS=(Interstitial Lung Diseases)) OR TS=(Diffuse Parenchymal Lung Diseases)) OR TS=(Interstitial Lung Disease)) OR TS=(Lung Disease, Interstitial)) OR TS=(Pneumonia, Interstitial)) OR TS=(Interstitial Pneumonia)) OR TS=(Interstitial Pneumonias)) OR TS=(Pneumonias, Interstitial)) OR TS=(Pneumonitis, Interstitial)) OR TS=(Interstitial Pneumonitides)) OR TS=(Interstitial Pneumonitis)) OR TS=(Pneumonitides, Interstitial)**

**[54,404](https://www.webofscience.com/wos/alldb/summary/db7d41ef-8ab2-47d8-85e6-e5b4b52b7205-04fbc45f/relevance/1)**

2

**(((((((((TS=(IFIH1 protein, human)) OR TS=(MDA-5 protein, human)) OR TS=(melanoma differentiation associated protein-5, human)) OR TS=(RH116 helicase, human)) OR TS=(MDA5 protein, human)) OR TS=(RNA helicase RH116, human)) OR TS=(anti-melanoma differentiation-associated gene 5 antibody)) OR TS=(anti-MDA5 antibody)) OR TS=(melanoma differentiation-associated gene 5)) OR TS=(MDA5)**

**[3,314](https://www.webofscience.com/wos/alldb/summary/25d50583-e6c0-4202-99b2-278bec7f1291-04fbbfdf/relevance/1)**

1

**(((((((((((TS=(dermatomyositis)) OR TS=(Dermatopolymyositis )) OR TS=(Polymyositis-Dermatomyositis)) OR TS=(Polymyositis Dermatomyositis)) OR TS=(Dermatomyositis, Adult Type)) OR TS=(Adult Type Dermatomyositis)) OR TS=(Dermatomyositis, Childhood Type)) OR TS=(Childhood Type Dermatomyositis)) OR TS=(Juvenile Dermatomyositis)) OR TS=(Dermatomyositis, Juvenile)) OR TS=(Juvenile Myositis)) OR TS=(Myositis, Juvenile)**

**[22,419](https://www.webofscience.com/wos/alldb/summary/4be246b0-8bcd-43c3-a8cf-36d8f9d34370-04fbb8ac/relevance/1)**
