# Supplementary figures and images for: Rituximab in the Treatment of Interstitial Lung Diseases Related to Anti-Melanoma Differentiation-Associated Gene 5 Dermatomyositis: A Systematic Review
Source: Front Immunol. 2022 Jan 18;12:820163. doi: 10.3389/fimmu.2021.820163 (PMC8803653; doi:10.3389/fimmu.2021.820163)

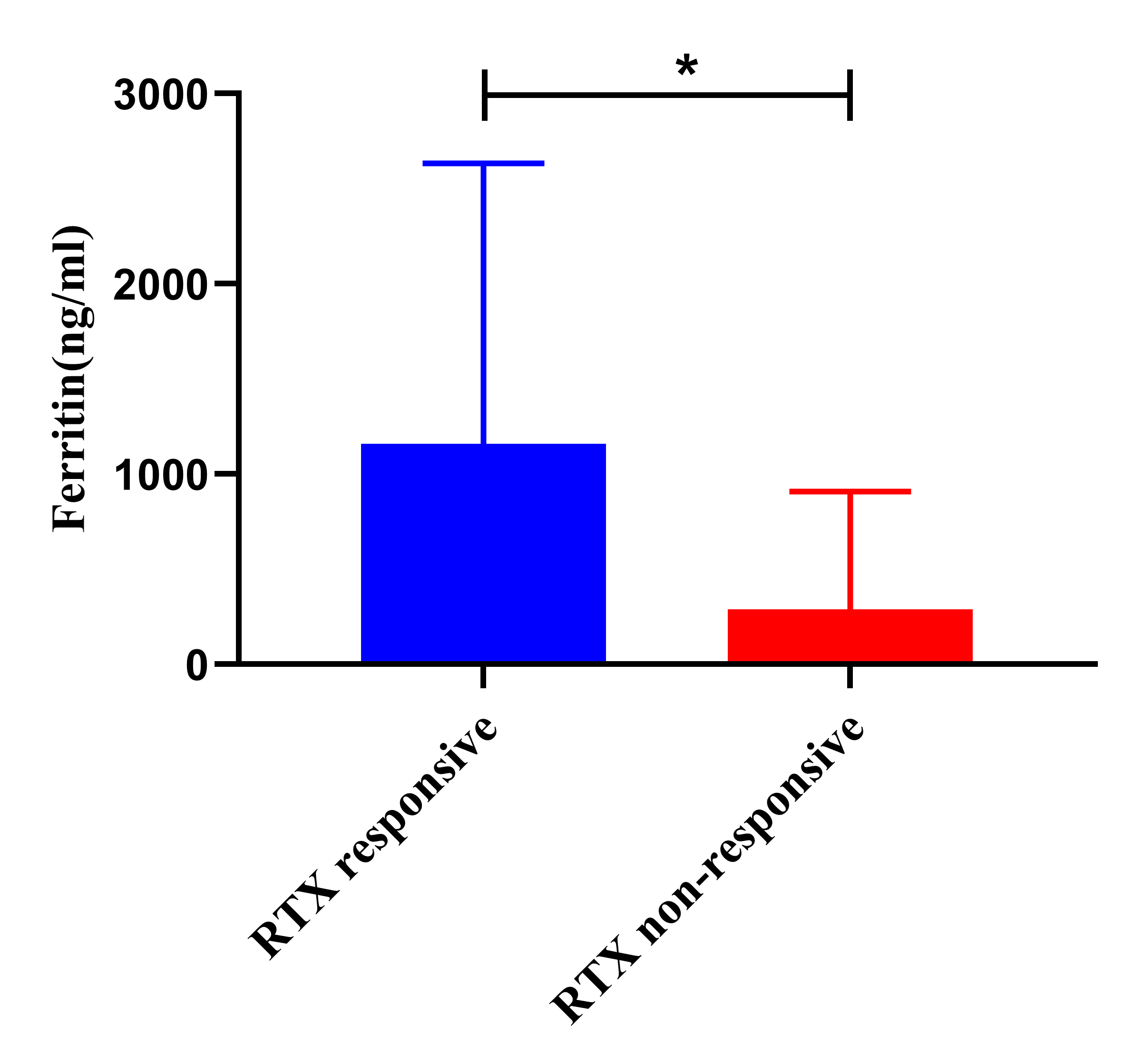

Supplement: Supplementary file 1 [file DataSheet_1.zip › Supplementary Figure 1.TIF]
